# Import of a major mitochondrial enzyme depends on synergy between two distinct helices of its presequence

**DOI:** 10.1042/BCJ20160535

**Published:** 2016-09-12

**Authors:** Ester Kalef-Ezra, Dimitra Kotzamani, Ioannis Zaganas, Nitsa Katrakili, Andreas Plaitakis, Kostas Tokatlidis

**Affiliations:** 1 Institute of Molecular Biology and Biotechnology, Foundation for Research and Technology, Heraklion (IMBB-FORTH), Crete, Greece; 2 Department of Biology, University of Crete, Heraklion, Crete, Greece; 3 Neurology Laboratory, School of Health Sciences, Faculty of Medicine, University of Crete, Heraklion, Crete, Greece; 4 Department of Materials Science and Technology, University of Crete, Heraklion, Crete, Greece; 5 Institute of Molecular Cell and Systems Biology, College of Medical Veterinary and Life Sciences, University of Glasgow, Glasgow G12 8QQ, U.K.

**Keywords:** glutamate dehydrogenase, human, mitochondria, presequence, protein import

## Abstract

Mammalian glutamate dehydrogenase (GDH), a nuclear-encoded enzyme central to cellular metabolism, is among the most abundant mitochondrial proteins (constituting up to 10% of matrix proteins). To attain such high levels, GDH depends on very efficient mitochondrial targeting that, for human isoenzymes hGDH1 and hGDH2, is mediated by an unusually long cleavable presequence (N53). Here, we studied the mitochondrial transport of these proteins using isolated yeast mitochondria and human cell lines. We found that both hGDHs were very rapidly imported and processed in isolated mitochondria, with their presequences (N53) alone being capable of directing non-mitochondrial proteins into mitochondria. These presequences were predicted to form two α helices (α1: N 1–10; α2: N 16–32) separated by loops. Selective deletion of the α1 helix abolished the mitochondrial import of hGDHs. While the α1 helix alone had a very weak hGDH mitochondrial import capacity, it could direct efficiently non-mitochondrial proteins into mitochondria. In contrast, the α2 helix had no autonomous mitochondrial-targeting capacity. A peptide consisting of α1 and α2 helices without intervening sequences had GDH transport efficiency comparable with that of N53. Mutagenesis of the cleavage site blocked the intra-mitochondrial processing of hGDHs, but did not affect their mitochondrial import. Replacement of all three positively charged N-terminal residues (Arg3, Lys7 and Arg13) by Ala abolished import. We conclude that the synergistic interaction of helices α1 and α2 is crucial for the highly efficient import of hGDHs into mitochondria.

## Introduction

Mitochondria have derived endosymbiotically 1.5–2 billion years ago [1] and ∼99% of mitochondrial proteins are encoded by nuclear DNA. These proteins are synthesized in the cytoplasm as precursors and are imported into the mitochondria via specific import pathways that direct them to one of the mitochondrial subcompartments: the outer membrane (OM), the inner membrane (IM), the intermembrane space (IMS) and the mitochondrial matrix.

The matrix-targeted polypeptides make up approximately two-third of the mitochondrial proteins [2]. They use the molecular channels translocase of the outer membrane (TOM) and translocase of the inner membrane 23 (TIM23) to cross the OM and IM, respectively [3], in a ΔΨ- and ATP-dependent manner. Most of the precursors of these proteins have a mitochondrial-targeting sequence (MTS) also called presequence [4,5]. The matrix presequences are found mostly at the N-terminus of the proteins and, in their majority, they consist of <50 amino acids [5–8] and their characteristics are quite distinct from the ER targeting peptides upon which the signal hypothesis was formulated [9]. The mitochondrial presequences are highly variable in their amino acid sequence; they tend to have a net positive charge and to be amphipathic in nature [7]. Following translocation into the mitochondria, these targeting peptides are typically removed by the mitochondrial matrix peptidase (MPP), a divalent metal ion-dependent protease in the mitochondrial matrix that recognizes a specific motif [10]. In addition, some matrix proteins require a second cleavage step for their maturation, which is effected by the intermediate cleaving peptidase 55 (Icp55) [11,12] or the octapeptidylpeptidase 1, also known as mitochondrial intermediate peptidase [13,14]. Although there are some common properties shared among most mitochondrial matrix-targeting presequences, the individual mechanistic properties of distinct peptide segments of longer and more complex presequences are not completely understood.

Mammalian glutamate dehydrogenase (GDH, EC 1.4.1.3) is a nuclear-encoded enzyme that catalyzes the reversible oxidative deamination of glutamate to α-ketoglutarate and ammonia, using NAD(H) and/or NADP(H) as cofactors. The enzyme is central to cellular metabolism by linking amino acid and carbohydrate metabolism and contributing to ammonia management, energy homeostasis and cell signalling [15–17]. This housekeeping enzyme (hGDH1 in the human) is encoded by the intron-containing glutamate dehydrogenase (human gene) (*GLUD1*) gene that is widely expressed. In addition, humans and other primates have acquired, by duplication of the *GLUD1* gene, the *GLUD2* gene encoding a highly homologous hGDH2 isoprotein that shows distinct functional properties and tissue distribution profile [18]. The biological importance of these proteins is highlighted by observations showing alterations in hGDH1 or hGDH2 in disorders of insulin homeostasis [19] and neurodegenerative diseases [20,21], and more recently in human glial tumours [22].

In mammals, the highest GDH activity is found in the liver where it localizes to all hepatocytes. In this organ, GDH is one of the most abundant proteins [1% (w/w) of proteins present in whole liver homogenate], constituting ∼10% of mitochondrial matrix proteins [23]. Krebs originally suggested that these huge enzyme levels are needed for keeping its reactants in equilibrium [24]. While GDH levels are lower in non-hepatic tissues characterized by cellular heterogeneity (brain, kidney and steroidogenic organs) [25], the enzyme can attain high levels (∼10 mg/ml of mitochondrial matrix) within individual cells that express this protein [23]. To maintain such high intra-mitochondrial levels, GDH depends on a very efficient mitochondrial transport system. In human cells, the mitochondrial import of hGDH1 and hGDH2 is mediated by an unusually long N-terminal cleavable presequence (N53) [26], deletion of which prevents the enzyme from entering the mitochondria [27,28]. The presequence of hGDH2 shows a higher degree of divergence compared with the mature protein. Thus, while the mature hGDH1 and hGDH2 differ in only 15 out of 505 of their amino acids, their presequences differ in 9 out of their 53 amino acids. Rosso et al. [29] have suggested that *GLUD2* evolution provided hGDH2 with enhanced mitochondrial-targeting capacity. The authors attributed this to a single evolutionary amino acid substitution (Glu7Lys) in the N-terminus of the MTS, which is thought to affect the positive charge of the peptide.

Here, we sought to elucidate the mechanisms by which hGDH1 and hGDH2 are imported into mitochondria. For this, we took into consideration the structural characteristics of the hGDH1 and hGDH2 mitochondrial presequences, thought to form two α-helices (α1 and α2) separated by loops. Our previous work has delineated the secondary structure elements and amphipathic nature of hGDH1 and hGDH2 presequences and has highlighted the essential role of the α1 helix [28]. Here, we sought to further characterize, in mechanistic detail the individual contributions of each of the two α-helices, the role of the net positive charge in the N-terminus and C-terminus of the presequence and the need for intra-mitochondrial cleavage. To this end, we combined mutagenesis studies, *in vitro* import assays using isolated yeast mitochondria, confocal microscopy in human cells as well as fractionation of cells stably expressing normal and mutant hGDH1 and hGDH2. We report that the mitochondrial import of hGDHs depends on the synergistic interaction of α1 and α2 helices and on the net positive charge of the presequences. Moreover, our data revealed that the intra-mitochondrial cleavage of the signal peptide is not a prerequisite for import.

## Experimental

### Materials

Media and reagents for cell culture and transfection were purchased from Gibco-BRL (Grand Island, NY, USA). Culture flasks, dishes and 6-well plates were purchased from Sarstedt AG & Co. Expression vectors *pEGFP-N3* and *pDsRed2-Mito* were obtained from BD Biosciences Clontech (Palo Alto, CA, USA). Paraformaldehyde (PFA) was obtained from Sigma Chemical Co. (St Louis, MO, USA). Fluorescence mounting medium was purchased from Dako (Carpinteria, CA, USA). Preparation of mitochondria from mammalian cells was performed using the Mitochondria Isolation Kit for Cultured Cells from Pierce Biotechnology (Rockford, IL, USA).

For *in vitro* radiolabelled protein synthesis, we used the TNT Coupled Reticulocyte Lysate System kit (Promega) and the appropriate plasmid constructs in pSP64 or pSP65 vectors (Promega). Primers were purchased from MACROGEN, and restriction endonucleases and T4 DNA ligase were purchased from MINOTECH Biotechnology (Heraklion, Crete) as well as from New England Biolabs, Inc. (Ipswich, MA, USA). The rabbit IgG anti-GFP polyclonal antibody was obtained from MINOTECH Biotechnology (702-2) and the rabbit anti-MnSOD polyclonal antibody from MERCK-MILLIPORE (06-984). The anti-Cpn10 (chaperonin 10) and the anti-Cytb2 (cytochrome b2) were a gift from Jeff Schatz's Laboratory. Visualization of protein bands following western blotting was performed with the SuperSignal West Pico Chemiluminescent Substrate (Thermo Fisher Scientific, Rockford) and the LumiSensor (GenScript).

For PCR, we used the DNA polymerases Taq (MINOTECH Biotechnology), One Taq (Biolabs) and Pfu Turbo (Stratagene). Purification of the PCR-amplified or digestion products was performed using the QIAprep Spin Miniprep kit (Qiagen) and the PCR Clean-up Gel Extraction kit (MACHEREY-NAGEL GmbH & Co.). The mutagenesis reactions were generated by PCR-based site-directed mutagenesis (Quick Change Site-Directed Mutagenesis kit; Stratagene).

### Methods

#### Cloning and mutagenesis

The cloning of GLUD1-EGFP, GLUD2-EGFP, Δ53GLUD1-EGFP, Δ53GLUD2-EGFP and Δα1GLUD2-EGFP (Δ38GLUD2-EGFP) in pEGFP-N3 has already been described [27,28]. All other constructs prepared for the present study are summarized in Supplementary Table S1. Primer design and the PCR conditions were performed in accordance with the manufacturer's instructions. The proper sequence and orientation of plasmid constructs were verified by bi-directional sequencing.

#### Import into yeast mitochondria

Precursors were synthesized in a rabbit reticulocyte lysate in the presence of ^35^S-methionine after *in vitro* transcription with SP6 polymerase. Import reactions were performed by incubating the reticulocyte lysate containing radiolabelled precursor at 30°C for 5 min (or the indicated time) in 50–100 μg wild-type (wt) *Saccharomyces cerevisiae* mitochondria in import buffer [2% (w/v) bovine serum albumin, 0.6 M sorbitol, 150 mM KCl, 10 mM MgCl_2_, 2.5 mM EDTA, 2 mM ATP, 2 mM NADH, 20 mM HEPES–KOH (pH 7.4)]. Where indicated, the potential across the mitochondrial inner membrane was dissipated with 2 μM valinomycin.

Mitochondria were then resuspended in 1.2 M sorbitol and 20 mM HEPES (pH 7.4), followed by a treatment with trypsin (0.1 mg/ml) with or without 1% (vol/vol) Triton X-100 (TX-100) for 30 min on ice to remove unimported material. Soybean trypsin inhibitor (1 mg/ml) was added to arrest the protease treatment. Finally, samples were resuspended in Laemmli sample buffer with β-mercaptoethanol, analyzed by SDS–PAGE and visualized by digital autoradiography (Molecular Dynamics) and processed in Adobe Photoshop CS5.

#### Preparation of mitoplasts from yeast mitochondria

To create mitoplasts (mitochondria without an intact outer membrane), the mitochondria were osmotically ruptured in a hypotonic buffer by dilution (after the import reactions) with 8 vols of 20 mM HEPES–KOH (pH 7.4) and incubation for 30 min at 4°C [30]. The samples were centrifuged at 16 000 ***g*** on ice for 5 min. Both the pellet and supernatant fractions were collected and precipitated with 10% trichloroacetic acid (TCA). Finally, samples were resuspended in Laemmli sample buffer with β-mercaptoethanol and analyzed by SDS–PAGE. Both the pellet and supernatant fractions were immunoblotted with antibodies.

#### Carbonate extraction of yeast mitochondria

After import reactions, mitochondria were resuspended in freshly made, ice-cold 0.1 M Na_2_CO_3_ and incubated on ice for 30 min [31]. The samples were centrifuged at 55 000 ***g*** on ice for 30 min. Both the pellet and supernatant fractions were collected and precipitated with 10% TCA, loaded on an SDS–PAGE and immunoblotted with antibodies.

#### Transient transfection and confocal microscopy

HEK293 (human embryonic kidney) and HeLa human cervical cancer (human cervical adenocarcinoma) cell lines were used for all transfection studies. The cells were maintained in Dulbecco's Modified Eagles Medium supplemented with 10% fetal bovine serum and gentamycin (50 μg/ml), at 37°C, under 5% CO_2_. For transfection, cells were seeded at 3 × 10^5^ cells/ml in a 6-well plate with a bottom coverslip, cultured for 24 h and transiently transfected with the recombinant plasmid DNA (3 μg/μl) using the calcium phosphate-mediated transfection procedure [32]. Co-transfection experiments were performed using a mixture of either the full-length plasmid wt *GLUD2-EGFP* or mutant plasmids and the mitochondrial targeting vector *pDsRed2-Mito*. Transfected cells were incubated at 37°C for 48 h, washed twice with phosphate-buffered saline (PBS) (pH 7.5), fixed with 4% PFA and mounted with Fluorescence mounting media for microscopy. The cells were examined (with sequential excitation at 488 nm for EGFP and at 568 nm for dsRED) by laser scanning confocal fluorescence microscopy (Leica TCS-NT Laser Scanning) using a ×10, ×40 or ×63 oil objective. Each co-transfection experiment was repeated three times. Co-localization of the *EGFP*-fusion plasmids with the *pDsRed2-Mito* was assessed by examining the transfected cells with ×10 objective. The images were acquired and merged using the Leica application software (Leica Microsystems Heidelberg GmbH). In certain cases, a ×2–8 digital zoom was used for better clarity. Images were exported as TIFF files and processed in Adobe Photoshop CS5.

#### Stable cell lines and cell fractionation

Stable HEK293 cells expressing hGDH1-EGFP, hGDH2-EGFP, N53(of hGDH1)-EGFP, N53(of hGDH2)-EGFP, α1-EGFP, α2-EGFP, α1α2-EGFP, α2α1-EGFP, Δ53hGDH2-EGFP, α1-Δ53hGDH2-EGFP and α1α2-Δ53hGDH2-EGFP were created as follows: the day before stable cell selection, cells were split in 35 mm dishes and transfected with the appropriate construct, using Lipofectamine 2000 Transfection Reagent (Invitrogen, Life Technologies). The following day, standard medium was replaced with medium containing G418, 600 μg/ml (Geneticin, Life Technologies), and the cells were left to grow for 1–2 weeks, until colonies of stable cells were visible. During the selection period, medium in the dishes was changed with fresh medium containing G418 (600 μg/ml) every 1–2 days. Selection efficiency (fluorescence signal) was monitored under an inverted fluorescence microscope. Colonies with most efficient selection were picked using a Gilson pipette with a sterile yellow tip and grown in 24-well dishes with 1 ml of media containing G418 in each well. Furthermore, subcloning to reach >95% homogeneity was performed.

For cell fractionation and preparation of mitochondria, only stable HEK293 cells were used. Stable cells were grown to 85–90% confluence in 100 mm dishes. Cells were harvested (∼2 × 10^7^ cells), washed once with PBS and centrifuged at 850 ***g*** for 2 min. Cell pellets were subsequently processed with the Mitochondria Isolation Kit for Cultured Cells, using the Dounce homogenization method, according to the manufacturer's instructions. Protein concentration was determined with the Bradford method, using the Bio-Rad Protein Assay (Bio-Rad Laboratories, USA). Mitochondrial and cytosolic fractions were run on a 10 or 12% SDS–PAGE. Proteins were transferred onto a nitrocellulose membrane and incubated with the anti-GFP antibody and the anti-MnSOD antibody (as a control of proper mitochondrial preparation).

#### Miscellaneous

Wt mitochondria were isolated from the *S. cerevisiae* strain D273-10B (MAT-α) as described previously [33]. The yeast strains were grown at 30°C in medium containing 1% (wt/vol) yeast extract, 2% (wt/vol) bacto-peptone and 2% lactate (vol/vol), pH adjusted to 5.5 (yeast peptone lactate (YPL)). SDS–PAGE was performed according to the standard procedures. For the separation of proteins below 15 kDa, Tris-Tricine SDS–PAGE was applied [34]. Western blots were performed on nitrocellulose membranes according to the standard procedures. In some figures, non-relevant gel lanes were excised by digital treatment. The web program *PredictProtein*, available at the electronic address http://www.predictprotein.org, was used to analyze the primary and secondary structure of hGDH presequences [35].

## Results

### Import of hGDH1 and hGDH2 by isolated mitochondria of *S. cerevisiae*

**Figure 1.**
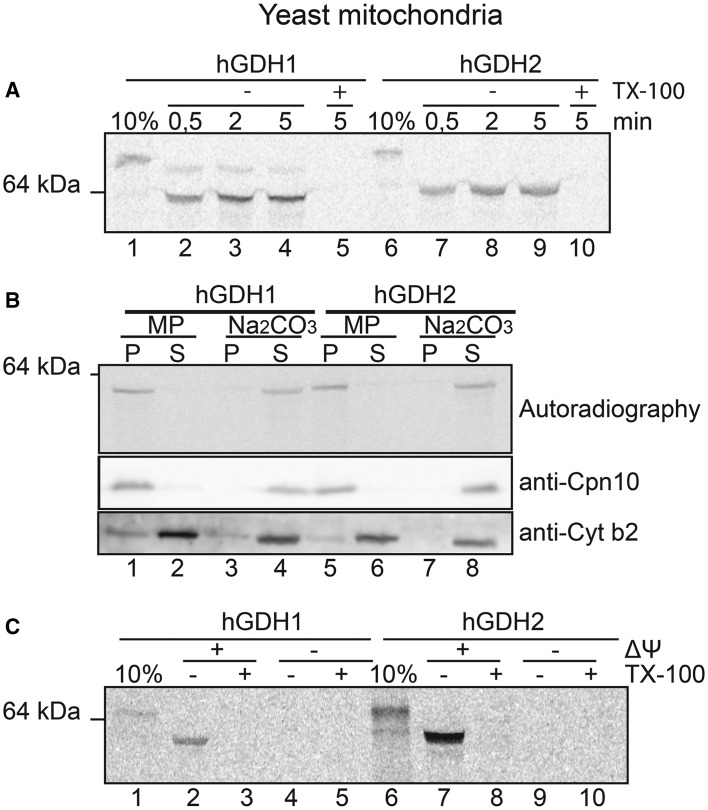
Human GDHs can be efficiently imported in yeast mitochondria and localize in the mitochondrial matrix in a ΔΨ and metal ion-dependent manner. Import of radioactive wt hGDH1 and hGDH2 in wt pure yeast mitochondria, monitored by SDS–PAGE and autoradiography, as described in Materials and Methods. (**A**) Import of hGDH1 and hGDH2 in mitochondria for 0.5 (lanes 2 and 7), 2 (lanes 3 and 8) or 5 (lanes 4, 5, 9 and 10) min. Ten per cent (lanes 1 and 6) represents the fraction of the total amount of *in vitro* translated material used for import. The + TX-100 samples (lanes 4 and 10) represent the samples that are extracted by TX-100 as a negative control for the mitochondrial import. (**B**) Import of hGDHs in mitochondria followed by subfractionation using MP (lanes 1 and 2 as well as 5 and 6) and carbonate extraction (Na_2_CO_3_, lanes 3 and 4 as well as 7 and 8). In all samples, trypsin was added at the end of the import reaction in intact mitochondria, which were then re-isolated by centrifugation prior to further treatment via MP or carbonate extraction. Upper panel shows the autoradiography result. The middle and the lower panels show western blot tests with anti-Cpn10 and anti-Cytb2 antibodies that were performed as control for the sub-mitochondrial fractionations. S, supernatant; P, pellet. (**C**) Import of hGDHs in yeast mitochondria with intact or disrupted electrochemical potential of the inner mitochondrial membrane, +ΔΨ or −ΔΨ, respectively.

The ability of wt hGDH1 or hGDH2 to translocate into the mitochondria was studied *in vitro* using mitochondria isolated from *S. cerevisiae.* Results revealed that *in vitro* synthesized, radiolabelled hGDHs were very rapidly imported and cleaved (within a few seconds) in isolated pure yeast mitochondria (Figure 1A).

This was confirmed by protease treatment, following solubilization of the mitochondrial membranes by TX-100. This treatment resulted in the degradation of the imported proteins (Figure 1A, ‘+TX-100’ lanes). Import kinetics showed a similar import rate for both human GDHs, with essentially all hGDH1 and hGDH2 used for import assays found in the mature form within 30 s after import (Figure 1A). However, a careful inspection of Figure 1A reveals that a faint band (migrating just below the unprocessed enzyme) is present above the mature hGDH1, but not above hGDH2. This may suggest a more rapid import and processing of hGDH2. Alternatively, the faint band may represent a tiny amount of synthesized hGDH1 that lacks three terminal amino acids (and therefore migrating just below the mature enzyme, as found in previous sequencing studies of GDH from human tissues) [18,36] and that may be processed a little slower than the full-length enzyme.

After import, we applied a mild osmotic shock to the mitochondria (mitoplasting, MP), which selectively ruptures the OM, but not the IM, releasing the contents of the IMS in the supernatant fraction. We found that both hGDHs were recovered from the resulting pellet that contains the intact IM (including the matrix) (Figure 1B). Additionally, sodium carbonate extraction followed by centrifugation (indicated as ‘Na_2_CO_3_’, Figure 1B), which releases soluble IMS and matrix proteins to the supernatant while leaving integral membrane proteins sedimenting to the pellet, showed that both hGDHs were in the supernatant (Figure 1B). These MP and carbonate extraction experiments, taken together, suggest that upon *in vitro* import in isolated yeast mitochondria, both hGDHs translocate into the mitochondrial matrix (Figure 1B). Finally, we found that the mitochondrial import of hGDHs was dependent on the mitochondrial membrane potential, as disrupting the inner membrane potential by carbonyl cyanide m-chlorophenyl hydrazine (CCCP) abrogated the import capacity of hGDHs into mitochondria (Figure 1C).

### Mitochondrial targeting analysis of the N53 presequence of human GDHs

**Figure 2.**
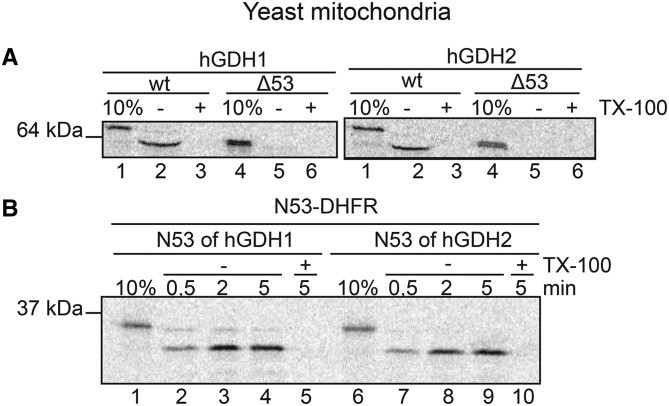
N53 is essential for the appropriate mitochondrial targeting of hGDHs and both hGDH presequences alone are able to target non-mitochondrial proteins in mitochondria. (**A**) Import of hGDH1 and hGDH2 (wt and Δ53hGDH) in yeast mitochondria for 5 min highlights the essential role of the N53 presequence in mitochondrial targeting of hGDHs. (**B**) Import of the N53-DHFR (of hGDH1) and N53-DHFR (of hGDH2) fusion proteins into mitochondria for 0.5, 2 or 5 min shows that both N53 presequences are highly capable of targeting the non-mitochondrial protein DHFR in yeast mitochondria.

We initially tested whether mature hGDH1 and hGDH2 that lack their N53 presequence can be imported into isolated mitochondria. Results revealed that deletion of the presequence (Δ53) rendered hGDHs unable to translocate into the isolated yeast mitochondria (Figure 2A), confirming previous observations based on transfection experiments in mammalian cell lines [27].

We then evaluated whether the hGDH1 and hGDH2 presequences could alone target non-mitochondrial proteins into mitochondria. To this end, we cloned the N53 of human GDH presequences to the N-terminal of the mouse non-mitochondrial proteins DHFR (dihydrofolate reductase) and EGFP (enhanced green fluorescent protein). Results revealed that the chimeric protein N53-DHFR (carrying the N53 of either hGDH1 or hGDH2) was indeed imported into and cleaved in yeast mitochondria in a time-dependent manner. Interestingly, the N53-DHFR of hGDH2 was processed faster than that of hGDH1 (Figure 2B), mirroring the results we obtained for full-length hGDH2 (Figure 1).This suggests that this behaviour divergence was due to the differences in the amino acid sequence of the two N53 signals rather than the structure of the mature proteins.

**Figure 3.**
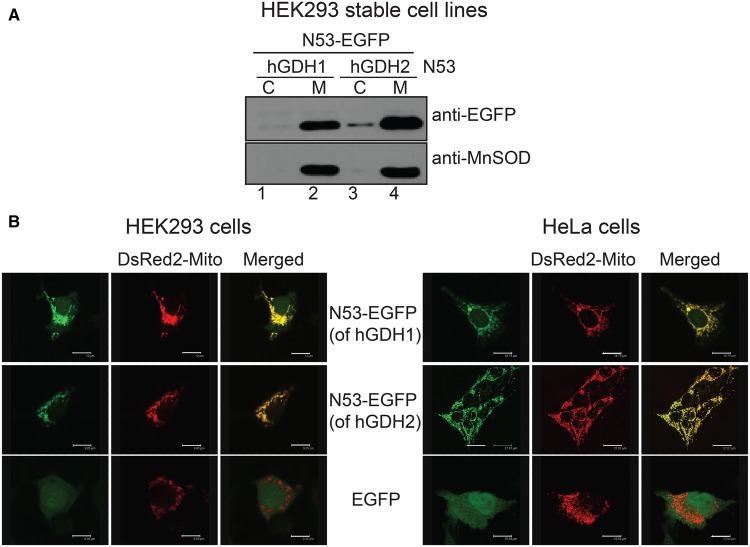
The N53 presequence of both hGDHs can target EGFP to mitochondria of HEK293 and HeLa cells. (**A**) Western blot analysis of N53-EGFP (of hGDH1 and hGDH2) expression in HEK293 cells, following transfection and generation of stable cell lines. Both N53 presequences, when fused to the N-terminal of EGFP, can be imported into the mitochondria, where they are detected in the M fractions (lanes 2 and 4). (**B**) HEK293 cells (left) or HeLa cells (right) were viewed by confocal microscopy, using a ×40 oil objective, 72 h following co-transfection with the N53-EGFP plasmids (of either hGDH1 or hGDH2) and with the DsRed2-Mito vector. N53-EGFP (of hGDH1) co-localizes with the mitochondrial marker (first row), as shown by the yellow colour in merged images (third column). Similarly, N53-EGFP (of hGDH2) shows co-localization with (second row). No distribution of green fluorescence outside of mitochondria is observed. EGFP alone is presented as a control (third row). The green fluorescence of EGFP protein is scattered throughout the cytosol and the cell nucleus, where it can be diffused due to its small size. Scale bars: HEK293 cells, first row: 12 μm, second row: 9.9 μm, third row: 9.4 μm; HeLa cells, first row: 18.8 μm, second row: 27.8 μm, third row: 15.5 μm.

We then created cell lines (HEK293) stably overexpressing N53-EGFP (including N53 of either hGDH1 or hGDH2) and performed subcellular fractionation and western blotting analysis of the resulting cytosolic and mitochondrial fractions. We observed that the fused N53-EGFP proteins (carrying the presequence of either hGDH1 or hGDH2) were recovered in the mitochondrial fraction (Figure 3A). We also studied the expression of the human GDH presequences fused with EGFP in HEK293 or HeLa cells using confocal microscopy. Transient co-transfection experiments with N53-EGFP (using the presequence of either hGDH1 or hGDH2) and DsRed2-Mito (mitochondrial marker) revealed that the fused N53-EGFP proteins co-localized with the mitochondrial marker (Figure 3B). Taken together, these results suggest that the presequence of hGDHs is sufficient to direct non-mitochondrial proteins into the mitochondria.

### Deletion of the predicted first α helix of N53 abrogates hGDH2 mitochondrial import

**Figure 4.**
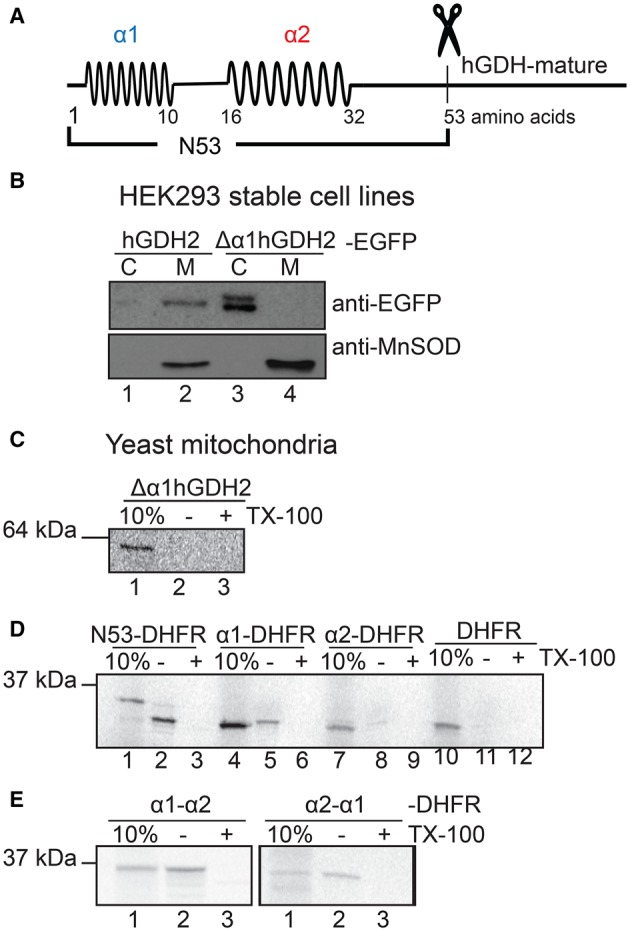
Both human GDH presequences form two distinct amphipathic α-helices (α1 and α2), and the α1 has an essential role in the mitochondrial import process. (**A**) Schematic representation of the structural properties of hGDH1 presequences. The presequence of both hGDHs are predicted to have the tendency to form two amphipathic α-helical structures, the α1 (blue colour) and the α2 (red colour). Scissors represent the position of their cleavage site. (**B**) Western blot analysis of hGDH2-EGFP and Δα1hGDH2-EGFP expression in HEK293 cells, following transfection and generation of stable cell lines. Tissue fractionation into mitochondrial (M) and cytosolic (C) fractions revealed that hGDH2 is present in the M fraction of HEK293 cells (lane 2), whereas Δα1hGDH2 that lacks the α1 helix cannot be imported into mitochondria and is detected in the C fraction (lane 3). Proper mitochondrial preparation was tested with an antibody against anti-MnSOD, a mitochondrial protein that is present only in the M fractions (lanes 2 and 4). (**C***–***E**) Import of radiolabelled Δα1hGDH2, N53-DHFR, α1-DHFR, α2-DHFR, α1α2-DHFR and α2α1-DHFR for 5 min in yeast mitochondria.

Secondary structure analyses of the presequences for hGDH1 and hGDH2 have suggested that both leader peptides are positively charged and that they form two to three α-helices, separated by intermediate loops. [28]. Our present analysis, based on prediction models (PredictProtein) [35], similarly predicts that although the N53 presequences of hGDH1 and hGDH2 differ in 9 out of their 53 amino acid residues (scattered throughout the entire presequence), they both include two distinct α-helices (α1: N 1–10; α2: N 16–32) separated by loops (Figure 4A).

Studies in HEK293 cells stably overexpressing hGDH2-EGFP or Δα1hGDH2-EGFP showed that the Δα1hGDH2-EGFP was also not imported into the mitochondria (Figure 4B). These results are in accordance with previously reported studies in HEK293, COS7 and SHSY-5Y cell lines [28]. Moreover, using the yeast mitochondria import system, we observed that deletion of the predicted first helix from the hGDH2 presequence (Δα1hGDH2) abrogated the import of radiolabelled hGDH2 into these mitochondria (Figure 4C).

### The mitochondrial-targeting function of individual α-helices of the hGDH2 presequence

**Figure 5.**
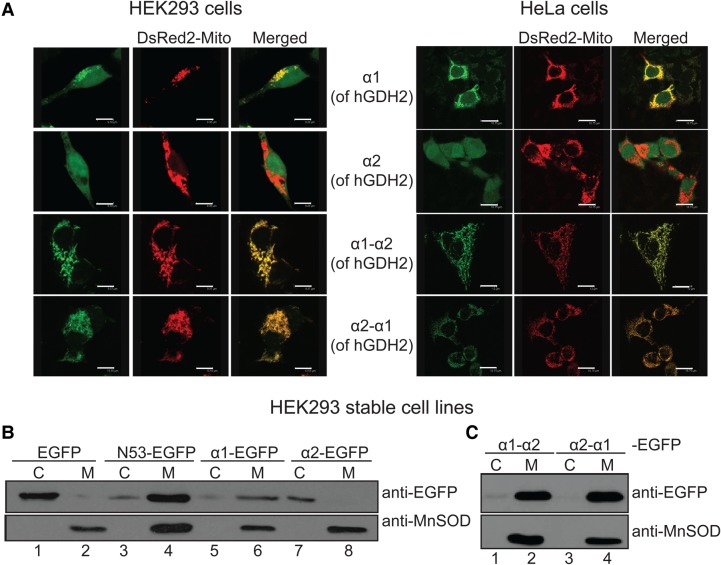
The α1 peptide of hGDH2 presequence, and not α2, is sufficient and capable of targeting EGFP in mitochondria of HEK293 and HeLa cells. (**A**) HEK293 cells (left) or HeLa cells (right) were viewed by confocal microscopy, using a ×40 oil objective, 72 h following co-transfection with the corresponding EGFP plasmids (α1-EGFP, α2-EGFP, α1α2-EGFP and α2α1-EGFP) and the DsRed2-Mito vector. First row: The α1-peptide is sufficient to target EGFP to mitochondria where it co-localizes with dsRed2-Mito, although a smaller amount of green fluorescence is detected in the cytosol and cell nucleus. Second row: The α2 helix is not capable of targeting EGFP to mitochondria. Green fluorescence of α2-EGFP is diffuse, including the cell nucleus; no co-localization with the dsRed2-Mito is observed. Third and fourth rows: Both α1α2-EGFP and α2α1-EGFP plasmids can be efficiently imported into the mitochondria of HEK293 and HeLa cells. Scale bars: HEK293 cells, first row: 9.4 μm, second row: 9.4 μm, third row: 9.4 μm; fourth row: 13.6 μm; HeLa cells, first row: 18.8 μm, second row: 18.8 μm, third row: 12 μm, fourth row: 19 μm. (**B**) Western blot analysis of EGFP, N53-EGFP (of hGDH2), α1-EGFP, α2-EGFP (left) and α1α2-EGFP, α2α1-EGFP (right) expression in HEK293 cells, following transfection and generation of stable cell lines. Tissue fractionation into mitochondrial (M) and cytosolic (C) fractions revealed that, while EGFP is localized in the cytosolic fraction (lane 1), N53-EGFP (of hGDH2) and α1-EGFP are detected predominantly in the M fractions (lanes 4 and 6). On the other hand, α2-EGFP is localized in the C fraction (lane 7) and not in the mitochondria (lane 8). (**C**) Moreover, α1α2-EGFP and α2α1-EGFP can be targeted into mitochondria of HEK293 cells (lanes 2 and 4).

In light of the above data, we then checked whether the predicted individual α-helices (α1 and α2) of the hGDH2 presequence can alone target non-mitochondrial proteins into mitochondria. To this end, we fused the α1 (residues 1–10) or α2 (residues 16–32) helical peptides of hGDH2 with the N-terminus of DHFR or the N-terminus of EGFP and performed *in vitro* and *in vivo* assays, respectively (Figures 4D and 5). We observed that only the α1 helical peptide, but not the α2, is sufficient to target efficiently either DHFR or EGFP to yeast or human mitochondria, respectively (Figures 4D and 5).

These findings were further confirmed by co-transfection experiments using the mitochondrial marker DsRed2-Mito (Figure 5A). Specifically, as shown in Figure 5A, the α1 peptide, when fused at the N-terminal of EGFP protein, it co-localized with the mitochondrial marker DsRed2-Mito, both in HEK293 and in HeLa cells. In contrast, when the α2 peptide was fused to EGFP, the combined protein failed to enter the mitochondria, (Figure 5B). Instead, the α2-EGFP fluorescence was distributed in the cytosol and in the cell nucleus (Figure 5B), in a pattern that resembles that of the EGFP alone (Figure 3B, lower panel).

To test whether the non-helical components of the hGDH2 presequence are essential for appropriate subcellular localization, we examined the mitochondrial-targeting ability of the α1α2 peptide of hGDH2 that lacks the predicted intermediate loop (residues 11–15) and the carboxyl end (residues 33–53), which includes the MPP recognition site (residues 50–51) and the cleavage site (residues 53–54). *In organello* as well as *in vivo* experiments showed that the α1α2 peptide, when fused N-terminally to DHFR or to EGFP, could be efficiently imported into mitochondria (Figures 4E and 5). However, as expected, the fused protein (lacking the MPP cleavage site) was not processed inside mitochondria (Figure 4E). Then, we checked the mitochondrial-targeting ability of the α2α1 peptide (constructed with two helical structures in reverse order) fused to DHFR or to EGFP. Results showed that the α2α1-fused proteins were also efficiently imported into the isolated mitochondria as DHFR or EGFP fused to the as α1α2-fused proteins did (Figures 4E and 5B). Finally, when α1 was fused to the C-terminal end of DHFR, it failed to transport DHFR into yeast mitochondria (results not shown).

**Figure 6.**
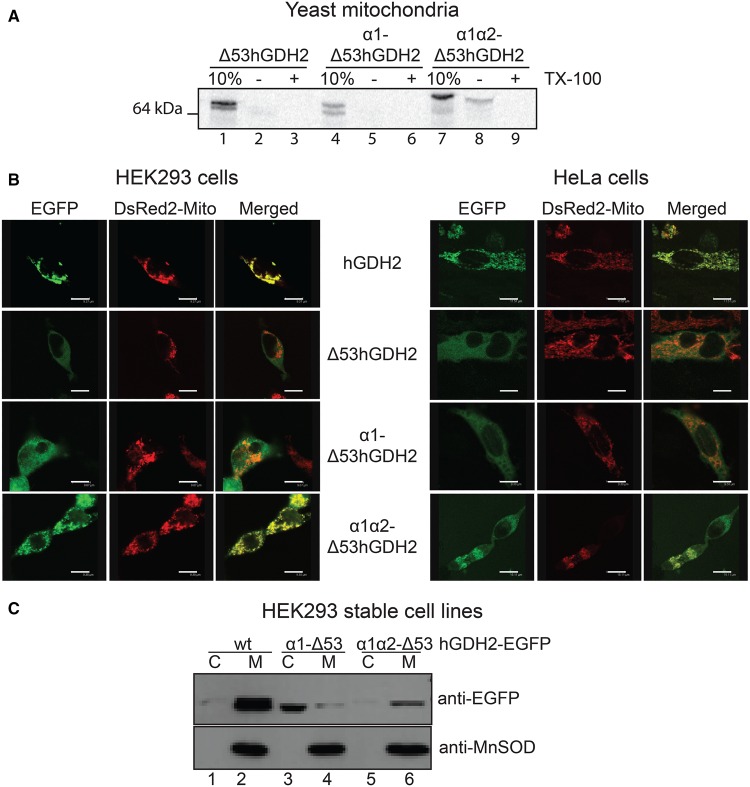
Both α1 and α2 are needed to target the mature hGDH2 into yeast and human mitochondria. (**A**) Import of the radiolabelled Δ53hGDH2 (lanes 2 and 3), α1-Δ53hGDH2 (lanes 5 and 6) and α1α2-Δ53hGDH2 (lanes 8 and 9) in yeast mitochondria and SDS–PAGE. (**B**) HEK293 cells (left) or HeLa cells (right) were viewed by confocal microscopy, following co-transfection with the EGFP plasmids and the DsRed2-Mito vector. First row: wt hGDH2-EGFP co-localizes with the mitochondrial marker. Second row: The Δ53hGDH2-EGFP shows a diffuse distribution of the green fluorescence in the cytosol. Third row: α1-Δ53hGDH2-EGFP cannot be targeted into mitochondria. Fourth row: The α1α2-Δ53hGDH2-EGFP co-localizes with the mitochondrial marker. Scale bars: HEK293 cells, first row: 9.4 μm, second row: 9.4 μm, third row: 9.7 μm, fourth row: 9.4 μm; HeLa cells, first row: 11.6 μm, second row: 10.3 μm, third row: 9.4 μm, fourth row: 15.1 μm. (**C**) Western blot analysis of α1-Δ53hGDH2-EGFP and α1α2-Δ53hGDH2-EGFP expression in HEK293 cells, following transfection and generation of stable cell lines. While wt hGDH2-EGFP is detected in the mitochondrial fraction (lane 2), α1-Δ53hGDH2-EGFP is detected predominantly in the cytosolic fraction (lane 3). On the contrary, α1α2-Δ53hGDH2-EGFP can be detected in the mitochondria of HEK293 cells (lane 6).

Although the α1 peptide was sufficient to guide the small non-mitochondrial proteins DHFR and EGFP into the mitochondria, it showed little capacity in directing the mature hGDH2 (Δ53hGDH2) protein into mitochondria. Thus, *in vitro* and *in vivo* assays in isolated yeast mitochondria and in human cell lines showed that α1-Δ53hGDH2 was not efficiently imported into mitochondria (Figure 6A–C). In contrast, the α1α2-Δ53hGDH2 was efficiently imported into yeast and human mitochondria (Figure 6A–C), indicating that both α1 and α2 are needed for the mitochondrial targeting of hGDH2. Moreover, the absence of the MPP cleavage site from this construct did not prevent this import (Figure 6A–C).

**Figure 7.**
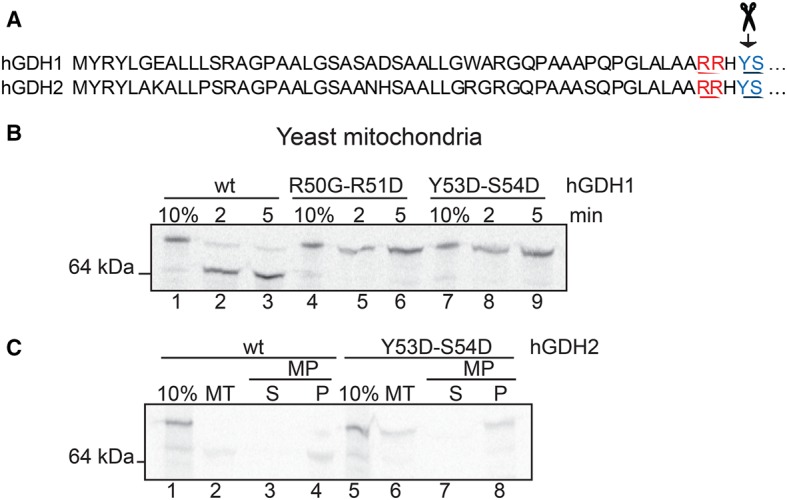
The cleavage in the mitochondrial matrix is not essential for mitochondrial targeting of hGDHs. (**A**) Schematic representation of the amino acid sequences of hGDH1 and hGDH2 presequences. The MPP recognition motif (R50-R51) is highlighted with red and the cleavage site (Y53-S54) of human GDHs is highlighted with blue. Scissors represent the position of the cleavage site of hGDHs. (**B**) Import of radioactive wt and double mutants of hGDH2 (R50G-R51D and Y53D-S54D) in wt yeast mitochondria for 2 or 5 min, monitored by SDS–PAGE and autoradiography. (**C**) Import of radiolabelled wt hGDH2 and hGDH2-Y53D-S54D in yeast mitochondria (MT, lanes 2 and 6) for 5 min. Additionally, import of radiolabelled wt hGDH2 and hGDH2-Y53D-S54D proteins in mitochondria following by subfractionation using MP and centrifugation (lanes 1 and 2 and 5 and 6). S, supernatant; P, pellet.

These findings are in agreement with results of mutagenesis experiments involving either the potential MPP recognition motif (Arg50–Arg51) or the cleavage site (Tyr53–Ser54), showing that these mutations did not impair the mitochondrial import of hGDHs, although they abrogated the cleavage of their presequence (Figure 7). Import experiments combined with MP showed that hGDH2-Tyr53Asp-Ser54Asp localized to the MP pellet as the wt hGDH2 did (Figure 7).

### Amino acid analysis and targeted mutagenesis

**Figure 8.**
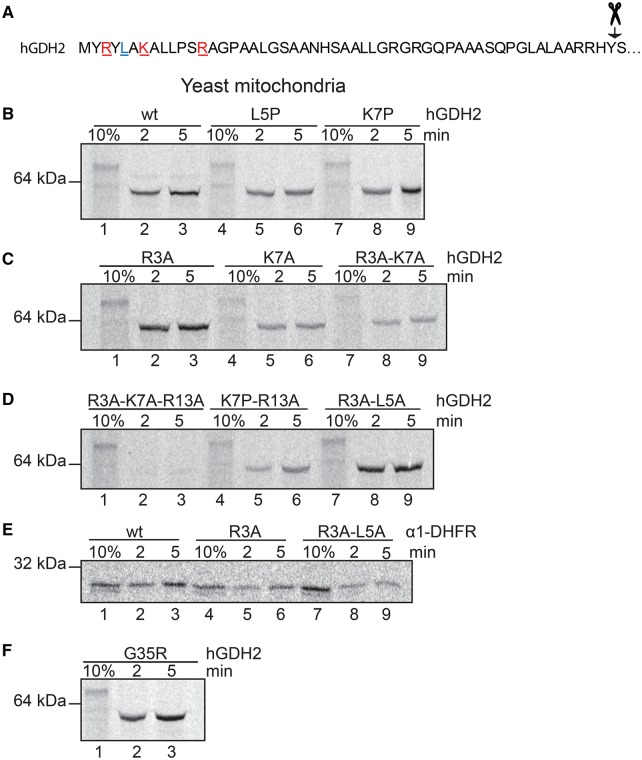
Import kinetic experiments of wt and mutated forms of hGDH2 and α1-DHFR (α1 of hGDH2) in yeast mitochondria. (**A**) The amino acid sequence of the hGDH2 presequence. The amino acids that were mutated in order to elucidate their role in mitochondrial import are highlighted with red and blue (red, positively charged; blue, neutral). (**B**–**D**) Import assays in yeast mitochondria of radiolabelled wt hGDH2 vs. mutated forms (L5P, K7P, R3A, K7A, R3A-K7A, R3A-K7A-R13A, K7P, R13A and R3A-L5A). All hGDH2 mutants can be efficiently imported into and cleaved in yeast mitochondria except from the triple mutant hGDH2-R3A-K7A-R13A (**D**, lanes 2 and 3). (**E**) Import assays in yeast mitochondria of radiolabelled wt α1-DHFR vs. mutated forms (R3A, R3A-L5A). (**F**) Import assays in mitochondria of the hGDH2-G35R that result from the common polymorphism c.G103A in the *GLUD2* gene.

As noted above, mitochondrial matrix signal peptides vary in their amino acid sequences although they share common characteristics, including a net positive charge, an amphipathic nature and a tendency to form α-helical structures. In light of these considerations, our next goal was to delineate the importance of the MTS biochemical characteristics in guiding hGDHs to the mitochondria. To this end, we performed site-directed mutagenesis in order to: (1) disrupt the α-helical configuration, (2) attenuate the positive charge of the presequence or (3) alter the amphipathic character of the first α-helix. Specifically, disruption of the α-helices was attempted by the insertion of prolines, which are known to act as structural disruptors when introduced in the middle of regular secondary structure elements such as α-helices and β-sheets. Since previous findings suggested that α1 is far more efficient than α2 for mitochondrial targeting, we disrupted the helical conformation of the α1 helix of hGDH2 through substitution of amino acids located in the middle of α1. Specifically, we replaced Leu5 or Lys7 in α1 by Pro (generating Leu5Pro or Lys7Pro mutants, respectively). Import assays showed that neither the hGDH2-L5P nor the hGDH2-K7P mutations affected the mitochondrial transport of the protein as the obtained mutants had a targeting efficiency that was similar to that of the wt hGDH2 (Figure 8B).

Additionally, we reduced, step by step, the positive charge of the N-terminal part of hGDH2 presequence by substituting Ala for Arg3 (Arg3Ala) or for Lys7 (Lys7Ala) and studied the mitochondrial import capacity of the obtained mutants. Results revealed that each of these single mutants could efficiently import the full-length hGDH2 into the mitochondria (Figure 8C). Then, we tested the hGDH2-Arg3Ala-Lys7Ala and hGDH2-Lys7Pro-Arg13Ala double mutants and found that both mutants could be imported into yeast mitochondria (Figure 8C,D). In contrast, replacing all three N-terminal basic amino acids (Arg3, Lys7 and Arg13) by Ala affected the mitochondrial-targeting capacity of the presequence. Thus, as shown in Figure 8D, the obtained triple mutant (hGDH2-Arg3Ala-Lys7Ala-Arg13Ala) was unable to enter the mitochondria, implying that the net positive charge of the N-terminal part of the hGDH2 presequence is crucial in this process. Finally, disruption of the amphipathic character of the α1 helix, by replacing Arg3 and Leu5 by Ala (Arg3Ala-Leu5Ala), did not affect the mitochondrial import of hGDH2 (Figure 8D).

Parallel experiments concerning the α1 peptide fused to DHFR (α1-DHFR) showed that the mutants α1-Arg3Ala-DHFR and α1-Arg3Ala-Leu5Ala-DHFR can be imported into yeast mitochondria, but with reduced efficiency when compared with the α1-DHFR (Figure 8E).

### Import analysis of the common polymorphism Gly35Arg of hGDH2

Previous work from our group revealed the existence of the single-nucleotide polymorphism c.G103A in the *GLUD2* gene, which results in the non-synonymous Gly35Arg change of the hGDH2 presequence. This polymorphism was found to be rather common as it was present in both Parkinson disease patients (16.7%) and in controls (18.3%). Moreover, its presence did not affect the phenotypic characteristics of Parkinson disease [21]. Here, we show that hGDH2 carrying the above change can be efficiently targeted to mitochondria (Figure 8F).

## Discussion

GDH is a very abundant mitochondrial protein (constituting ∼10% of matrix proteins) in several mammalian cell types, where it is thought to play a central role in metabolic and other cellular processes. The ability of this nuclear DNA-encoded enzyme to accumulate in huge amounts in the mitochondrial matrix suggests a highly efficient mitochondrial import system that has not been well understood. Previous studies by Mihara et al. [37], using reticulocyte lysates, revealed that GDH synthesized by this cell-free translation system was of larger molecular weight than the mature protein found in mammalian tissues. The authors suggested that mammalian GDH is synthesized as a precursor protein that undergoes processing inside the mitochondria to generate the mature GDH [37]. This hypothesis was confirmed by the cloning of the *GLUD1* [26] and *GLUD2* [38] genes, which allowed the prediction that a 53 amino acid-long N-terminal peptide not present in the endogenous hGDHs served as a mitochondrial leader signal. However, previous ^35^S pulse-chase experiments in human hepatoma HepG2 and glioma U373 cell lines detected a single GDH isoprotein after 5 min of chase, the molecular weight of which was identical with that of mature human brain GDH [36]. While the reasons for not detecting even a trace of precursor GDH generated by these cell lines within 5 min of chase had remained unclear, our present observations provide a rational explanation as detailed below.

Here we sought to elucidate the mechanisms by which human GDH1 and GDH2 translocate into the mitochondria by employing both human cell lines and isolated yeast mitochondria, to draw a comprehensive picture of the import pathway. The yeast mitochondrial import system offers several advantages over the use of the mammalian system, as it is evolutionarily conserved from yeast to humans and it is generally reproducible and relatively easy to use. Results revealed that both human isoproteins are rapidly transported and processed in isolated yeast mitochondria, with the entire process requiring <30 s. Hence, the present study not only uncovered a highly efficient mitochondrial transport system that permits GDH to accumulate in huge amounts in the mitochondrial matrix, but it also provided a rational explanation for the inability of the previous pulse-chase experiments to detect GDH precursors synthesized by the cells studied [36].

To further characterize the mitochondrial import machinery used by hGDH1 and hGDH2, the presequence of which shows a greater degree of divergence than the mature enzymes, we performed kinetic analysis of the processing of the two human enzymes in isolated mitochondria. Although these revealed a faster maturation of hGDH2 compared with hGDH1, we could not distinguish whether the proteolytic process is faster for hGDH2 or whether there is a difference in kinetics in the import process itself. Since both hGDHs share the same MPP processing site, the difference in proteolytical cleavage observed here may relate to differential recognition by other mitochondrial peptidases, like Icp55 (Icp55 kDa). Icp55 cleaves one (or three amino acids) from an MPP-generated intermediate, generating a more stable protein, in a two-step cleavage mechanism [11,12,39]. The presence of +3L in hGDH2 (+3A in hGDH1) may facilitate recognition and processing by Icp55, leading to an overall faster presequence elimination. Icp55 has been so far characterized mainly in yeast, even though homologues may exist in mammalian organisms [40]. On the other hand, when studied alone in the absence of the mature protein, the hGDH2 presequence (N53) is still processed faster than the hGDH1 presequence. This suggests that faster processing may depend on faster import of hGDH2, due to its enhanced mitochondrial targeting. Further work will have to be undertaken to investigate in detail this hypothesis in human cells.

In light of the above findings, it is of importance to identify the structural elements of the hGDH1 and hGDH2 presequences that ensure rapid translocation of these proteins into mitochondria and understand the biological need for the unusually large size (N53) of these presequences. While mitochondrial presequences are known to be highly variable in their primary structure, they share some common biochemical properties. These include the presence of positively charged, hydrophobic and/or hydroxylated amino acid residues and the tendency to form amphipathic α-helices [5]. Consistent with this model, our previous work, based on *in silico* methodology, has shown that human GDH presequences are predicted to form amphipathic α-helices (ref. [28]) and that the α1 helix is essential for hGDH2 mitochondrial targeting (ref. [28]). This previous study, however, raised the question on the role of the α2 helix. Here, we analyzed comprehensively the contributions of the two helices in detail. We provide evidence that both α1 and α2 helices need to co-operate, in a synergistic manner, for efficient targeting of the mature proteins into mitochondria. Thus, while selective deletion of the α1 helix abrogated the mitochondrial import of hGDH1 and hGDH2 (ref. [28] and our Δα1 data), this helix by itself displayed little, if any, mitochondrial-targeting capacity for the human enzymes. On the other hand, peptides consisting of only the α1 and α2 helices without the intermediate loops could restore the mitochondrial targeting of the mature protein. Remarkably though, the α1 helix alone could direct efficiently non-mitochondrial proteins (DHFR and EGFP) into mitochondria. This could be explained by the different size of the proteins (hGDH2: 56 kDa; DHFR: 22 kDa; EGFP: 27 kDa) and/or by the net charge of the amino acid sequence downstream of the presequence in the mature part of the proteins.

Our study showed that the N-terminal part of the hGDH presequences (containing the α1 helix and the three positively charged amino acids, Arg3, Lys7 and Arg13) has a critical role in mitochondrial import. However, ornithine transcarbamylase (OTC) relies on the mid-Arg-rich portion (N8–22) of its 32 amino acid-long presequence [41]. On the other hand, the relatively short presequence (N19) of the rat liver aldehyde dehydrogenase (ALDH) relies on two small α-helices (4–10,14–19) for its mitochondrial-targeting capacity as deletion of either helix abrogated the import of the precursor protein [42,43]. Moreover, deletion of a three-residue linker segment that connects the two α-helices increased the helix-forming ability and import competence [41]. In contrast, our data showed that removal of the connecting loops of hGDH presequences had no effect on mitochondrial import.

Although the above two paradigms emphasize the role of the positively charged residues (OTC) or of small helical structures (ALDH) within the presequence, the 53 amino acid-long leader peptide of hGDHs may have provided a biological advantage by possessing both distinct helical structures and a net positive charge. Specifically, the net positive charge of the mitochondrial-targeting presequence of hGDH2 may provide a molecular explanation for the efficiency of mitochondrial targeting of this enzyme in glial cells where the mitochondrial inner membrane potential is lower than in other cell types like hepatic cells [44]. In this context, it is noteworthy that the α1-helix has a higher net charge than the α2-helix, which is in agreement with a more prominent role of α1 in mitochondrial targeting to the organelle.

Most of the proteins that are targeted into the mitochondrial matrix of eukaryotes have an N-terminal presequence that is proteolytically cleaved upon import into mitochondria. This process is thought to help proteins to stabilize and attain their proper structure in the mitochondrial matrix. In contrast with this expectation, we report here that amino acid replacements that abolish processing of the hGDH presequence (Arg50Gly-Arg51Asp and Tyr53Asp-Ser54Asp) do not affect the mitochondrial import capacity of mutated hGDHs, although it prevented the intra-mitochondrial cleavage of the presequence. These results imply that proteolytic cleavage of the presequence is not essential for mitochondrial import of human GDHs, although it is not known whether unprocessed hGDH proteins can obtain their appropriate hexameric structure and can be enzymatically active. In this regard, there are paradigms of mitochondrial targeted proteins that do not process a cleavable presequence, including the GDH of *Arabidopsis thaliana* [45], the rat Cpn-1 and the pig mitochondrial aspartate aminotransferase [46,47].

The above findings raise important questions as to whether mitochondrial processing plays a role by permitting the protein to go to other cellular compartments, either directly or following a retrograde movement causing export from mitochondria as shown for pyruvate dehydrogenase (PDH) [48] or yeast fumarase [49]. Although mammalian GDH1 has been previously used as a mitochondrial marker, it was recently shown that hGDH1 also localizes to the nucleus of some astrocytes and of the vast majority of human cortical oligodendrocyte precursors [50]. Because oligodendrocyte precursors are known to differentiate to oligodendrocytes and to some of the protoplasmic astrocytes, and because dividing cells require higher levels of intracellular α-ketoglutarate levels than differentiated cells [51], the presence of hGDH1 in the nucleus of these cells may be of importance for the function of α-ketoglutarate-dependent nuclear dioxygenases known to be involved in histone and DNA modification processes [51]. Similarly, PDH, once it translocates from the mitochondria to the nucleus, is thought to supply acetyl CoA for histone acetylation [48]. In light of these considerations, additional studies are needed to elucidate whether the independence of intra-mitochondrial processing from the mitochondrial import of hGDHs, as observed here, serves additional functions for these remarkable proteins.

In conclusion, our study revealed a complex interplay between the two distinct α-helical regions and the importance of the positive charge of the unusually long presequence of GDH. The present results provide important insights into the mechanisms that govern the mitochondrial transport of a major mitochondrial matrix protein and, as such, they have important implications for a more thorough understanding of the intracellular trafficking and subcellular sorting of nuclear-encoded proteins synthesized in the cytosol.

## Abbreviations

ALDH, aldehyde dehydrogenase; CCCP, carbonyl cyanide m-chlorophenyl hydrazine; Cpn10, chaperonin 10; Cytb2, cytochrome b2; DHFR, dihydrofolate reductase; EGFP, enhanced green fluorescent protein; GDH, glutamate dehydrogenase; GLUD1, glutamate dehydrogenase (human gene); HEK, human embryonic kidney; HeLa, human cervical adenocarcinoma; Icp55, intermediate cleaving peptidase 55; IM, inner membrane; IMS, intermembrane space; MP, mitoplasting; MPP, mitochondrial matrix peptidase; MTS, mitochondrial-targeting sequence; OM, outer membrane; OTC, ornithine transcarbamylase; PBS, phosphate-buffered saline; PDH, pyruvate dehydrogenase; PFA, paraformaldehyde; TCA, trichloroacetic acid; TIM23, translocase of the inner membrane 23; TOM, translocase of the outer membrane; TX-100, Triton X-100; wt, wild type; ΔΨ, mitochondrial inner membrane potential; YPL, yeast peptone lactate.

## Author Contribution

E.K.-E. designed and performed the experiments in the yeast system. D.K. designed and performed the experiments in human cell lines. E.K.-E. and D.K. analyzed the results, prepared the figures and contributed most of the text in the manuscript. I.Z. co-ordinated the study and analyzed the results. N.K. provided technical assistance and contributed to the mitochondrial isolation from yeast. A.P. and K.T. conceived and co-ordinated the study, analyzed the results and reviewed and edited the final version of the paper. All authors discussed the results and commented on the manuscript.

## Funding

This work has been financed by the European Union (European Social Fund — ESF) and Greek national funds through the Operational Program ‘Education and Lifelong Learning’ of the National Strategic Reference Framework (NSRF) — Research Funding Program: THALIS — UOC, Title ‘Mitochondrial dysfunction in neurodegenerative diseases’ [Grant Code 377226]. Work in K.T.’s laboratory was partly funded by the Scottish Universities Life Sciences Alliance-Scottish Funding Council (SULSA) [SULSA-SFC HR07019], the Royal Society (Wolfson research merit award [grant code WM120111]), the Wellcome Trust Institutional Strategic Support Funds [grant code 097821/Z11/Z] and the Research Funding Program: ARISTEIA-IMBB ‘Mechanisms of mitochondrial oxidative protein folding in biogenesis, physiology and disease’ [grant code 148].
